# *In Situ* Activation of Pituitary-Infiltrating T Lymphocytes in Autoimmune Hypophysitis

**DOI:** 10.1038/srep43492

**Published:** 2017-03-06

**Authors:** Han-Huei Lin, Angelika Gutenberg, Tzu-Yu Chen, Nu-Man Tsai, Chia-Jung Lee, Yu-Che Cheng, Wen-Hui Cheng, Ywh-Min Tzou, Patrizio Caturegli, Shey-Cherng Tzou

**Affiliations:** 1 Department of Biological Science and Technology, National Chiao Tung University, Hsin-Chu 30068, Taiwan; 2 Department of Neurosurgery, Johannes Gutenberg University, Mainz 55131, Germany; 3 Institute of Molecular Medicine and Bioengineering, National Chiao Tung University, Hsin-Chu 30068, Taiwan; 4 Department of Medical Technology and Biotechnology, School of Medical Laboratory and Biotechnology, Chung Shan Medical University, Clinical Laboratory, Chung Shan Medical University Hospital, Taichung 40201, Taiwan; 5 Department of Biochemistry and Molecular Genetics, University of Alabama at Birmingham, Birmingham, AL 35294, USA; 6 Department of Pathology, Johns Hopkins Medical Institutions, Baltimore, MD 21205, USA

## Abstract

Autoimmune hypophysitis (AH) is a chronic inflammatory disease characterized by infiltration of T and B lymphocytes in the pituitary gland. The mechanisms through which infiltrating lymphocytes cause disease remain unknown. Using a mouse model of AH we assessed whether T lymphocytes undergo activation in the pituitary gland. Infiltrating T cells co-localized with dendritic cells in the pituitary and produced increased levels of interferon-γ and interleukin-17 upon stimulation i*n vitro*. Assessing proliferation of CD3- and B220-postive lymphocytes by double immunohistochemistry (PCNA-staining) and flow cytometry (BrdU incorporation) revealed that a discrete proportion of infiltrating T cells and B cells underwent proliferation within the pituitary parenchyma. This proliferation persisted into the late disease stage (day 56 post-immunization), indicating the presence of a continuous generation of autoreactive T and B cells within the pituitary gland. T cell proliferation in the pituitary was confirmed in patients affected by autoimmune hypophysitis. In conclusion, we show that pituitary-infiltrating lymphocytes proliferate *in situ* during AH, providing a previously unknown pathogenic mechanism and new avenues for treatment.

Autoimmune hypophysitis (AH) is an inflammatory disease of pituitary gland^1^, typically manifesting with headache, visual disturbances, and various degrees of hypopituitarism. AH can be fatal if the adrenal insufficiency is unrecognized^2^,^3^, so that some cases are still reported at autopsy^4^ (see Table 1 in ref. 4). AH is characterized pathologically by an infiltration of the pituitary gland with a plethora of hematopoietic cells, mainly lymphocytes and plasma cells^5^, which initially lead to expansion (causing headache and visual disturbance) and ultimately atrophy of the pituitary (causing hypo-pituitarism^6^). Other than the demonstration of pituitary-infiltrating lymphocytes, the pathogenesis of AH remains unknown. Consequently, the treatment options for AH are limited. AH is typically treated symptomatically with glucocorticoids, a treatment that is associated with high recurrence rate^7^. Recent evidence also suggests that surgery to remove inflamed pituitary tissue and decompress the sella turcica is also not able to prevent recurrences^7^.

AH can occur spontaneously without identifiable causes (primary AH), or be caused by the administration of cancer immunotherapies (secondary AH). In the latter group, the greatest number of patients have been reported after treatment with monoclonal antibodies directed against cytotoxic T lymphocyte antigen-4 (CTLA-4)^8^,^9^. CTLA-4 is a molecule mainly expressed on T lymphocytes that normally inhibits T cell activation and proliferation. Therefore, when CTLA-4 is blocked T cells become more active and capable of destroying tumor cells that normally escape their surveillance. The downside of this enhanced T cell activity is that CTLA-4 blockade also triggers a wide range of autoimmune side effects, collectively referred to as immune-related adverse events (irAEs). The most common irAEs are dermatitis, colitis, hepatitis, and hypophysitis^10^,^11^,^12^. The incidence of hypophysitis induced by CTLA-4 blockade is now estimated to be around 11%. For example, Faje *et al*. recently reported that 17 of 154 melanoma patients developed hypophysitis after the CTLA-4 blockade therapy^13^. Other immune checkpoint inhibitors, such as antibodies directed against PD-1 or PD-L1, can also cause hypophysitis but at a much lower incidence^14^,^15^.

AH was first reported in 1962 in its primary form^16^ and in 2003 in the form secondary to CTLA-4 blockade^10^. Despite these early reports, however, the detailed pathogenic mechanisms underlying AH remain unknown^5^,^17^,^18^. Few reports have thus far attempted to investigate the pathologies of autoimmune hypophysitis^5^,^17^,^18^. These papers focused on the histochemical delineation of cell types and the activation status of the infiltrating cells that may present in the pituitary sections of hypophysitis patients. Despite these studies, how infiltrating T cells and B cells contribute to disease progression is largely unclear. A better understanding into its pathogenesis should benefit the management of autoimmune hypophysitis patients.

Lack of an appropriate animal model and the inability to identify the autoantigens in autoimmune hypophysitis has hampered our understanding of this disease. To address these shortcomings, we previously developed a mouse model of autoimmune hypophysitis based on the immunization of female SJL mice with whole mouse pituitary proteins emulsified in complete Freund’s adjuvant (CFA)^19^,^20^, a model that recapitulates many pathological, hormonal and radiological features of the human counterpart. We then identified growth hormone as a target recognized by pituitary-infiltrating B cells, and found that immunization with purified recombinant mouse growth hormone induced a disease similar to that induced by whole pituitary proteins (Tzou SC *et al*., manuscript in preparation). In this study, we used the growth hormone immunization model to investigate the pathogenesis of autoimmune hypophysitis. Our results show that T cells infiltrating the mouse pituitary gland proliferate and secrete interferon-γ (IFN-γ) and interleukin-17 (IL-17). A similar phenotype, albeit to a lesser extent, was found in T cells infiltrating the pituitary gland of patients affected by autoimmune hypophysitis. These data reveal a previously unknown pathogenic mechanism in autoimmune hypophysitis and may be relevant to the human disease.

## Results

### Pituitary-infiltrating T cells co-localized with dendritic cells in the pituitary gland of mice that developed experimental autoimmune hypophysitis

**Figure 1:**
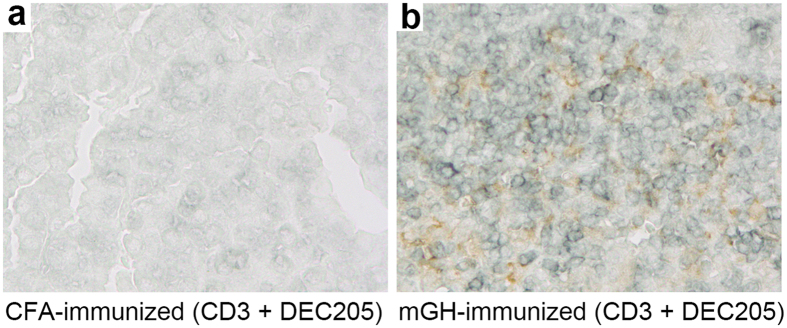
Co-localization of dendritic cells and T cells in the pituitary gland of mouse autoimmune hypophysitis. Pituitary sections from (**a**) CFA-immunized mice and (**b**) mGH-immunized mice were immunostained by DEC205 (in brown) and CD3 (in blue). Note dendritic brown staining in close proximity to blue staining in (**b**).

**Figure 2:**
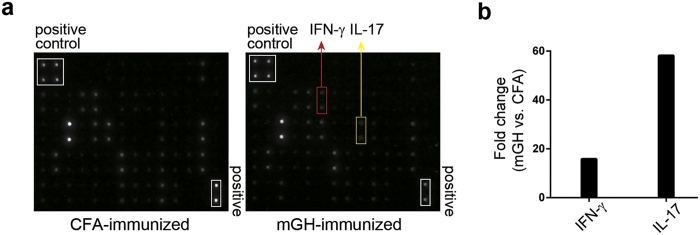
Heightened productions of IFN-γ and IL-17 by pituitary-infiltrating cells in mouse autoimmune hypophysitis. Single-cell suspensions from the pituitaries of CFA-immunized mice or mGH-immunized mice were stimulated by mGH. Cytokine productions after stimulation were detected by cytokine array membranes. The dots that represent IFN-γ and IL-17 on the membrane are indicated (**a**). Signal intensity of IFN-γ and IL-17 was measured for the two groups of mice. Differences in the cytokine level, expressed as fold change (mGH vs. CFA), were derived by dividing the signal intensity of mGH group by the signal intensity of the CFA group (**b**).

In the hypophysitis model induced by immunization with whole pituitary proteins, we noted considerable numbers of pituitary dendritic cells (CD11c^+^) cells in close proximity to lymphocyte aggregates (unpublished observation), suggesting that dendritic cells present antigens to pituitary-infiltrating T cells. Here, we first tested whether dendritic cells and T cells also co-localize in the pituitary glands of mice immunized with growth hormone. Double immunostaining for CD3^+^ T cells and DEC205^+^ dendritic cells indeed revealed co-localization (Fig. 1), suggesting ongoing interaction between these two cell types in the inflamed pituitary. A known outcome of this interaction is the activation of T cells and secretion of cytokines. To assess this outcome in the pituitary gland, we prepared pituitary single cell suspension from mice immunized with mouse growth hormone or CFA only, and stimulated the cells *in vitro* with mouse growth hormone. Cytokine secretion in culture supernatants was then detected by cytokine arrays. We found that IFN-γ and IL-17 were more strongly produced by T cells isolated from growth hormone-immunized mice than by cells isolated from control CFA-immunized mice (Fig. 2a). In particular, IFN-γ secretion was 15.8-fold higher and IL-17 secretion 58.2-fold higher in growth hormone cases than CFA controls (Fig. 2b). Although T cells have been reported to express the receptor for growth hormone^21^, our findings of increased IFN-γ and IL-17 secretion do not likely result from direct signaling from the mouse GH added to the cell cultures because cytokine secretion from the pituitary cultures was significantly more intense than from splenocytes, in both growth hormone-immunized mice and CFA-immunized mice (Supplementary Figure 1). Other differentially expressed cytokines or chemokines included IL-3 (2.6-fold), MIG (2.5-fold) and TCA-3 (2.6-fold) (Supplementary Figure 1). IL-6 was highly produced by single cell suspensions of both experimental groups (Supplementary Figure 1), a finding likely not secondary to contamination with bacterial products such as LPS, considering that the same immunogens did not stimulate IL-6 secretion from splenocytes of both groups (Supplementary Figure 1). Overall, these results suggest that T cells are activated by antigen presenting cells in the mouse pituitary gland to secrete inflammatory cytokines.

### Pituitary-infiltrating T and B cells proliferated in the pituitary gland of mice with experimental autoimmune hypophysitis

**Figure 3:**
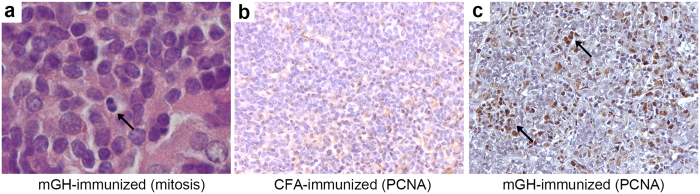
Heightened cellular proliferation in the pituitary gland of mouse autoimmune hypophysitis. Pituitary section from an mGH-immunized mouse showing a cell in the anaphase of the cell division (arrow) (**a**). Pituitary sections from CFA-immunized (**b**) and mGH-immunized mice (**c**) were immunostained for cell proliferation marker PCNA (in brown). Note numerous brown nuclear staining and small clusters of proliferating cells (arrows) in (**c**).

Co-localization with dendritic cells and cytokine secretions implies that pituitary-infiltrating T cells respond to antigens processed by antigen presenting cells in the inflamed pituitary. One of the early outcomes of this response is the proliferation of the activated T cells. Indeed, we identified mitotic cells in pituitary sections from mice that developed autoimmune hypophysitis (Fig. 3a). When immunostained for proliferating cell nuclear antigen (PCNA), a protein expressed by cells that are actively duplicating DNA prior to cell division, pituitary sections from mice that developed experimental autoimmune hypophysitis showed more proliferating cells (Fig. 3c) than CFA-immunized controls (Fig. 3b). Most of the PCNA positive cells morphologically resembled hematopoietic mononuclear cells and seemed restricted to the pituitary parenchyma, suggesting *in situ* proliferation. In some cases, proliferating cells aggregated to form small clusters in the pituitary (arrows in Fig. 3c). In the pituitary of mice that received CFA-only injection, only relatively few proliferating endocrine cells were noted, possibly representing homeostatic proliferation of pituitary cells^22^. Thus, autoimmune hypophysitis is accompanied by heightened cellular proliferation of infiltrating hematopoietic cells.

**Figure 4:**
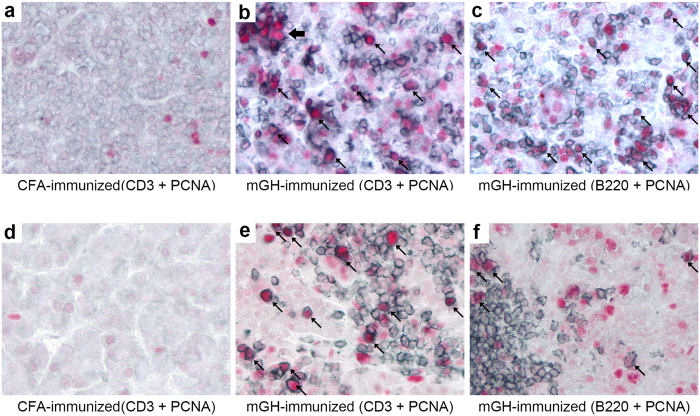
Persistent T and B cell proliferation in the pituitary gland of mouse autoimmune hypophysitis. Pituitary sections of mice that develop autoimmune hypophysitis on day 28 (**b** and **c**) or day 56 (**e** and **f**) were immunostained by PCNA (in red) and CD3 (**b** and **e**, in blue) or B220 (**c** and **f**, in blue). Proliferating cells are indicated by arrows. Note a small cluster of T cells that were stained by PCNA (thick arrow) in (**b**). Pituitary sections from CFA-immunized mice were immunostained for PCNA and CD3 as a negative control (**a** and **d**).

We next double immunostained mouse pituitary sections with antibodies to PCNA and lymphoid markers, CD3 for T cells and B220 for B cells, to identify the cell type that divided in the pituitary. In mice that developed hypophysitis after growth hormone immunization, infiltrating T cells could be readily identified in the pituitary (blue surface staining, Fig. 4), either scattered throughout the gland or focally clustered. Many of the CD3 positive T cells were also stained positive for PCNA (arrows, Fig. 4b), suggesting local proliferation rather than migration from the peripheral lymphoid organs of previously dividing T cells.

B cells also infiltrated the mouse pituitary after growth hormone immunization, although to a lesser extent, and comprised a subset of proliferating cells (arrows, Fig. 4c). These data demonstrate that in mouse autoimmune hypophysitis T cells and B cells proliferated *in situ* following infiltration of the pituitary parenchyma.

### Proliferation of T and B lymphocytes persisted in mid-late stage of mouse autoimmune hypophysitis

We have previously shown that mouse autoimmune hypophysitis can last for months after disease initiation^19^. We then assessed whether the proliferation of infiltrating lymphocytes seen in the early disease stage persisted into later stages. To this end, we immunized mice with recombinant mouse growth hormone and sacrificed mice at day 56 or day 63 post-immunization, a time point that we have previously shown overt endocrine deficits develop in these mice. Similar to published results^19^, histological examination of these pituitary glands revealed clear lymphocytic infiltration (Supplementary Figure 2). To examine whether infiltrating T cells and B cells were proliferating in the mouse pituitaries in the later stage of autoimmune hypophysitis, we immunostained the pituitary sections from mid-late disease for PCNA, plus CD3 or B220. PCNA positive T cells (arrows, Fig. 4e) and, to a lesser extent, B cells (arrows, Fig. 4f) could also be found in these late disease pituitary sections, with frequencies similar to those observed in the early (day 28) disease stage. Therefore, the proliferation of infiltrating T cells (and B cells) did not seem to diminish over time.

Multinucleated giant cells have been reported in our previous studies of autoimmune hypophysitis induced by mouse pituitary whole proteins (Fig. 5E in ref. 19). Multinucleated giant cells were also found in pituitary sections obtained from mice that developed mid-late stage hypophysitis immunized by mouse growth hormone. Interestingly, these multinucleated giant cells were also PCNA positive (Supplementary Figure 3). Whether they play a pathogenic role in mouse autoimmune hypophysitis remains unclear, but they may become stimulated in response to interferon-γ secreted by activated T cells^23^. Collectively, these data indicate that sustained proliferation of pituitary-infiltrating T and B cells may contribute to chronic inflammation of the pituitary.

### Flow cytometric analyses on the proliferating T and B lymphocytes in the pituitary gland of mouse autoimmune hypophysitis

Although PCNA is used as a marker of DNA synthesis, it can be also expressed in response to DNA damages. In addition, immunohistochemistry is a qualitative but not a quantitative analysis. To better characterize cellular subsets of proliferating cells and enumerate the proliferating pituitary-infiltrating T and B cells, we measured bromodeoxyuridine (BrdU) incorporation by flow cytometry. Mice immunized with recombinant mouse growth hormone for 28 days were injected BrdU 4 hours before sacrifice. This limited exposure time was chosen to minimize the inclusion of lymphocytes egressing from peripheral lymph nodes that had recently undergone proliferation. Single cell suspension from the pituitaries were prepared and sequentially stained for cell markers and intracellular BrdU for flow cytometric analyses.

**Figure 5:**
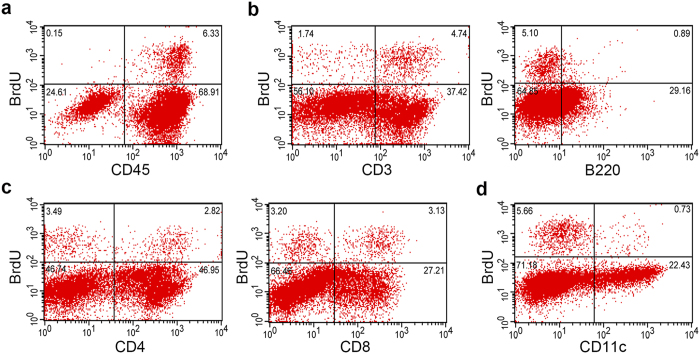
Flow cytometric analyzes of proliferating cells in the pituitary gland of mouse autoimmune hypophysitis. mGH-immunized mice were intraperitoneally injected with BrdU 4 and 2 hours before sacrifice on day 28 post-immunization. Cellular subsets that incorporated BrdU were analyzed by pan-leukocyte marker CD45 (**a**), lymphoid markers CD3 and B220 (**b**), markers for T cell subsets CD4 and CD8 (**c**) and CD11c (**d**). Representative data of two independent experiments are shown.

When single cells were gated on the pan-leukocyte marker CD45 and BrdU incorporation, the majority of proliferating cells were leukocytes (CD45^+^), consistent with our immunohistochemical findings (Fig. 5a). Of the infiltrating leukocytes, approximately 9% [6.3%/(6.3% + 68.91%)] were proliferating in the pituitaries of mice developed hypophysitis. Pituitary stromal cells (CD45^−^) represented only a small fraction (0.6%) [0.15%/(0.15% + 24.61%)] of BrdU^+^ cells (Fig. 5a). Analysis of the lymphoid subsets revealed that approximately 11% of CD3^+^ T cells and 3% of B220^+^ B cells incorporated BrdU (Fig. 5b). Although CD8^+^ T cells were less frequent than CD4^+^ T cells in the infiltrates, a higher frequency [10.3%; 3.13%/(3.13% + 27.21%)] of CD8^+^ T proliferated in the pituitary than the CD4^+^ T cells [5.67%; 2.82%/(2.82% + 46.95%)] (Fig. 5c). Finally, we also found that a small population (3%) of CD11c^+^ cells, possibly dendritic cells and macrophages, stained positive for BrdU (Fig. 5d). A summary of percentage of proliferating cells in each cell type is provided in Supplementary Table 2.

### T cell proliferation in the pituitary gland of patients with autoimmune hypophysitis

**Figure 6:**
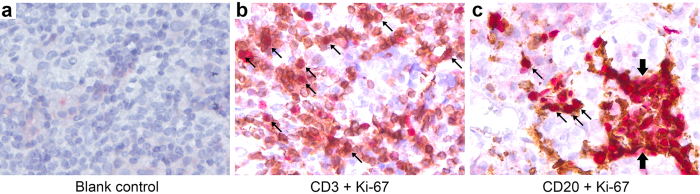
T and B cell proliferation in the pituitary gland of human autoimmune hypophysitis. Pituitary sections of human hypophysitis patients were immunostained by secondary antibody (blank control, (**a**)) or by Ki-67 (in red) and CD3 (in brown, (**b**)) or CD20 (in brown, (**c**)). Proliferating cells are indicated by arrows. Note a cluster of B cells that were stained positive for Ki-67 (thick arrows) in (**c**).

We then extended our mouse findings to humans by double immunostained human pituitary sections taken from autoimmune hypophysitis patients with Ki-67, a marker associated with cellular proliferation, and CD3 (for T cells) or CD20 (for B cells). In most patients, the lymphocytic infiltration was much milder than that observed in mice, except for a woman that was diagnosed with hypophysitis during the third trimester of pregnancy. Her pituitary showed conspicuous infiltration with T and B cells that were actively proliferating (Fig. 6). Although the sample size of this case series is small, our data suggest that infiltrating lymphocytes undergo *in situ* proliferation also in the pituitary gland of patients with autoimmune hypophysitis.

## Discussion

In the current study, we aimed to study the pathogenesis of autoimmune hypophysitis in greater depth using a recently identified autoantigen (growth hormone) in the mouse model. Our results show that dendritic cells co-localized with some T cells in the inflamed pituitary glands. In autoimmune hypophysitis, dendritic cells may acquire autoantigens released from the dead pituitary cells and present these autoantigens to activate the pathogenic T cells. In line with this view, we have previously demonstrated that the majority of pituitary-infiltrating T cells displayed activated/memory surface phenotypes^19^. This notion is further supported that *in vitro* stimulation of the pituitary-infiltrating cells by mouse growth hormone increased IFN-γ and IL-17 productions. Another important event following T cell activation by antigen presenting cells is the proliferation of these cells. Indeed, we observed a considerable fraction of T cells and B cells were proliferating in the mouse pituitary glands, persisting from early stage to mid-late stage of the disease. Lymphocyte proliferations were also confirmed in the pituitary gland of human autoimmune hypophysitis. Proliferation of pituitary-infiltrating T cells and B cells therefore may contribute to a continuous output of autoreactive T and B cells in the pituitary and sustain the chronic inflammation.

It is commonly held that activation and proliferation of naïve T cells occur in secondary lymphoid organs^24^. Once activated, T cells migrate to target sites to perform their effector functions. In this paradigm, effector T cell proliferation in the target sites is not considered necessary for their functions^25^. However, several studies have emerged to suggest that in some cases, T cells do proliferate in target tissues. For example, T cell proliferation was reported in the joints of human rheumatoid arthritis patients^26^ and in the pancreatic islets of NOD mice^27^,^28^. Furthermore, virus-specific memory T cells were shown to proliferate in the HSV-infected ganglia^29^,^30^. Finally, active immunization of OVA peptides to mice induces proliferation of OVA-specific T cells in the inflamed footpads^31^. A common theme in these studies is the chronic inflammation or the persistence of antigens in the target sites. Thus, antigen presenting cells (particularly the dendritic cells) can take up antigens to activate antigen-specific T cells *in situ* in the chronically inflamed tissues. Although myelin-specific T cells do not seem to proliferate in the CNS in experimental autoimmune encephalomyelitis^32^,^33^, they require re-stimulation by meningeal antigen presenting cells to enter the CNS parenchyma^34^,^35^. Thus a secondary activation in the inflammatory tissues seems a common requirement for at least some autoimmune diseases to progress, and blocking this secondary activation may be an effective strategy to treat autoimmune diseases.

Autoimmune hypophysitis is often induced by the CLTA-4 blocking antibody, ipilimumab, in melanoma patients. The mechanistic links between ipilimumab and hypophysitis remain unclear. Our group recently demonstrated that CLTA-4 blocking antibodies can damage CTLA-4-expressing endocrine cells in the pituitary and induce a mild lymphocytic infiltration^36^. On the other hand, CLTA-4 blocking antibodies may act directly on the pituitary-specific T cells. As CTLA-4 blockade is known to enhance T cell proliferation in the periphery^37^, it is tempting to speculate that blocking CTLA-4 can promote T cell proliferation in the pituitary. Furthermore, CTLA-4 blockade in T cells increases cytokine production (such as IL-17)^38^,^39^, thus aggravating disease progression. Accordingly, one rational therapeutic strategy for autoimmune hypophysitis is to block dendritic cell-T cell interaction in the pituitary by CTLA-4Ig (a fusion protein of the extracellular domain of CTLA-4 and an Fc fragment of IgG), given that our data show that T cells are activated in the pituitary. The latter is significant because treatment option is currently limited for this disease. Thus our results provide a tentative mechanism of how CTLA-4 blockade induces autoimmune hypophysitis and may point to a new venue of therapy development for autoimmune hypophysitis.

Mouse pituitary-infiltrating cells expressed interferon-γ and IL-17 after stimulation by growth hormone. IFN-γ and IL-17 can only be produced by the pituitary-infiltrating lymphocytes since single cell suspension isolated from the pituitaries of CFA-immunized mice (which did not develop any sign of lymphocytic infiltration) did not produce these two cytokines. Consistent with our finding, IFN-γ- and IL-17-positive T cells have been identified in the pituitary gland of patients with autoimmune hypophysitis^18^. Both IFN-γ and IL-17 are involved in the pathogenesis in other well-known autoimmune diseases such as thyroiditis, multiple sclerosis and type I diabetes. While interferon-γ may worsen^40^,^41^,^42^ or improve disease progression^43^,^44^, pathogenic role of IL-17 is well-established for many autoimmune diseases^45^,^46^,^47^. IL-17 may thus represent an attractive molecular target for treating autoimmune hypophysitis in the future, as some therapies targeting IL-17 pathways have been shown to benefit patients afflicted by autoimmune diseases^48^,^49^. Further studies are required to delineate the pathogenic roles and therapeutic potentials of IL-17 and IFN-γ in autoimmune hypophysitis.

We did not observe as extensive T and B cell proliferation in the pituitaries of human patients as in the mouse model, except in a female patient that presented autoimmune hypophysitis during her late pregnancy. The discrepancy between mouse model and human patients may be due to the heterogeneity of human patients such as association to pregnancy and age. It would be important to determine whether higher level of lymphocyte proliferation is found in classical cases of autoimmune hypophysitis (females associated with pregnancy), since the majority of female patients were diagnosed autoimmune hypophysitis at late pregnancy or early post-partum. In line with the current findings, it was reported that pregnant patients (including the pregnant patient reported here) showed most abundant CD8^+^ T cells that displayed activated phenotype (granzyme B^+^) in the pituitaries^5^. On the other hand, it should be noted that some of our patients were treated by glucocorticoids (Supplementary Table 1), which is known to suppress cell proliferation^50^,^51^. Finally, T cell proliferation is known to decrease with aging^52^, may explain lower lymphocyte proliferation in the pituitaries of the elder patients included in this study. Further studies using larger numbers of patients (especially female patients who are associated with pregnancy) are useful in addressing this question. If proven correct, this observation indicates that inhibition of secondary T cell activation/proliferation may be a plausible way to treat autoimmune hypophysitis in patients.

In conclusion, we provide evidences that T cells are activated to proliferate and secrete cytokines in the pituitary gland of autoimmune hypophysitis. These findings provide a previous unrecognized pathogenic mechanism and may point new avenues for further studies in this increasingly recognized disease.

## Methods

### Autoimmune hypophysitis patients

Pituitary samples from 8 patients with autoimmune hypophysitis were obtained at transsphenoidal surgery during the 1996–2008 period. Relevant clinical data on these patients are presented in Supplementary Table 1. All the patients gave informed consent before surgery. All immunohistochemical studies were approved, and performed in accordance with the standards set forth by the ethics committee of the Georg August University (18/10/03).

### Mice

SJL/J mice were purchased from the Jackson Laboratory (Bar Harbor, ME, U. S. A.) and bred in the animal facility of the National Chiao Tung University. For maintaining the genetic background of the SJL/J strain, male and female SJL/J mice were purchased once a year from the Jackson Laboratories and added to the breeding scheme. Female SJL/J mice were used in the study at 8-to-10 weeks old. All experiments were approved and conducted in accordance with the standards established by the National Chiao Tung University Animal Care and Use Committee.

### Production of recombinant mouse growth hormone

The DNA fragment encoding the mature mouse growth hormone (without signal peptide) was PCR amplified from a mouse cDNA template (IMAGE 30250975, Open Biosystems, Huntsville, AL, U. S. A.), and cloned into the pSMT3 vector (kindly provided by Dr. Jin-Biao Ma, Department of Biochemistry and Molecular Genetics, University of Alabama at Birmingham) at the BamHI and XhoI sites. pSMT3 is a T7 promoter-driven prokaryotic expression vector that encodes an N-terminally histidine-tagged SUMO protein, which serve as a fusion partner to promote solubility of recombinant proteins in bacterial cytoplasm. The DNA construct was sequenced to confirm the absence of mutations and the in-frame fusion between SUMO protein and mouse growth hormone. An additional serine between SUMO and mGH was introduced due to the restriction sequences needed to clone mouse growth hormone into this vector.

SHuffle T7 Express (NEB, Ipswich, MA, U. S. A.) competent *E. coli* cells were transformed with pSMT3-mGH and grown in ZYM505 medium^53^ supplemented with 20 μg/ml of kanamycin (Sigma-Aldrich, St. Louis, MO, U. S. A.) at 30 °C with vigorous shaking for overnight. For large-scale expression, *E. coli* cells (300 ml of overnight culture) were centrifuged at 5000 *g*, then re-suspended and grown at 20 °C for 24 hours with vigorous shaking in three liters of ZYM-505^53^. Production of the SUMO-mGH recombinant protein was induced by 1 mM IPTG (Invitrogen, Carlsbad, CA, U. S. A.) at 26 °C for six hours. The cells were harvested, washed in 50 mM Tris pH 8.0, and lyzed first by 0.1 mg/ml lysozyme (Sigma-Aldrich) in 50 mM NaCl, 50 mM Tris, 100 nM PMSF, pH 8.0. After stirring for 30 minutes, Triton-X100 (1%, Sigma-Aldrich) and DNase I (10 units/ml, Sigma-Aldrich) was added for additional 30 minutes. Cleared lysate, obtained by centrifugation at 25,000 *g*, was added to a 20 mM final concentration of imidazole (Sigma-Aldrich) and loaded onto a nickel-IDA agarose column (Sigma-Aldrich). Bound mGH was successively washed with one bed volume (BV) of binding buffer (150 mM NaCl, 50 mM Tris pH8, 0.1% Triton-X 100 and 20 mM of imidazole), one BV of washing buffer (binding buffer with 500 mM NaCl), one BV of glycerol washing buffer (binding buffer with 10% glycerol) and 10 BV of detergent-free binding buffer (binding buffer without Triton-x100). Sumo-mGH fusion protein was eluted with 250 mM imidazole in detergent-free binding buffer, and dialyzed at 4 °C in 25 mM phosphate buffer, pH 8.5 containing 0.01% sodium azide. To cleave SUMO from the fusion protein, SUMO protease (LifeSensors, Malvern, PA, U. S. A.) was added in the last change of dialysis buffer and incubated for two days. Digested proteins were further dialysis in 25 mM phosphate buffer pH 8.0 at 4 °C overnight. SUMO proteins and uncleaved SUMO-mGH and SUMO proteases (all contain histidine tags) were adsorbed to a nickel-IDA agarose column in 20 mM of imidazole, while cleaved growth hormone (no histidine tags) eluted from the column. Purified mouse growth hormone was further purified to near homogeneity by a single run of S-100 size-exclusion chromatography (GE Life Sciences, Marlborough, MA, U. S. A.).

### Induction of autoimmune hypophysitis in mice

Purified recombinant mouse growth hormone (mGH, 5 mg/mL) was emulsified 1-to-1 in CFA (which contained 5 mg/mL of heat-killed *Mycobacterium tuberculosis*, strain H37Ra, from BD Diagnostic Systems, Sparks, MD, U. S. A.), and injected subcutaneously on day 0 in a volume of 100 μl (50 μL in the dorsal hind leg region and 50 μL in the contralateral inguinal region). Protein emulsions were injected again on day 7 in the opposite sites. Mice were sacrificed at 28 (early stage) or 56 (mid-late stage) days post-immunization.

### Pituitary histopathology

Pituitary glands from mGH-immunized (N = 7) and CFA-immunized (N = 4) mice were dissected, fixed overnight in saline-buffered formalin, processed, and embedded in paraffin. At least five non-consecutive sections (1 every 10) were cut, stained by hematoxylin and eosin, mounted and observed with a light microscope.

### Pituitary single immunohistochemistry

Pituitary sections were deparaffinized and rehydrated. Antigen retrieval was performed in heated 10 mM Tris, 1 mM EDTA buffer. After blocking with nonspecific binding (by 10% normal goat serum), pituitary sections were stained by rabbit anti-PCNA antibodies (1:2000, Abcam, Cambridge, MA, U. S. A.). After proper wash and block for endogenous peroxidases by 0.3% H_2_O_2_, sections were incubated with biotinylated goat anti-rabbit IgG (1:2000, Jackson ImmunoResearch Laboratories, West Groove, PA, U. S. A.), followed by streptavidin-HRP (1:500, Dako, Glostrup, Denmark). Staining of PCNA in cells was revealed by addition of DAB solution (Invitrogen). Stained sections were counterstained by hematoxylin, mounted and observed under a light microscope.

### Pituitary double immunohistochemistry

For detecting the co-localization of T cells and dendritic cells in the inflamed pituitaries, sections were first incubated with rabbit anti-DEC205 antibodies (1:200, Abcam) plus rat anti-CD3 (1:40, AbD Serotec, Oxford, UK). CD3 staining was revealed by sequential addition of ImmPRESS HRP anti-Rat Ig and ImmPACT SG peroxidase substrate (all from Vector Labs, Burlingame, CA, U. S. A.). Sections were then treated 3% H_2_O_2_ to inactivate ImmPRESS HRP anti-Rat Ig. DEC205 staining was revealed by sequential addition of biotinylated goat anti-rabbit IgG streptavidin-HRP and DAB substrates. Stained sections were mounted and observed under a light microscope.

For detecting the type of lymphocytes that was proliferating in the inflamed pituitaries, sections were first incubated with rat anti-CD3 (AbD Serotec) or rat anti-B220 (1:100, eBioscience, San Diego, CA, U. S. A.), plus rabbit anti-PCNA antibodies. The sections were then incubated with ImmPRESS HRP anti-Rat Ig or ImmPRESS-AP anti-Rabbit Ig kits (Vector Labs). Antigen labeling was revealed by sequentially adding ImmPACT SG peroxidase substrate (blue-to-grey, for cell type labeling, from Vector Labs) and then Vector Red alkaline phosphatase substrate (red, for PCNA labeling, from Vector Labs). Stained sections were mounted and observed under a light microscope.

For detecting proliferation of T cells and B cells in the pituitaries of human hypophysitis patients, formalin-fixed, paraffin-embedded hypophysitis specimens were used. Antigen retrieval by microwave cooking in EDTA solution was applied to the sections for all stainings. To detect T cell or B cell proliferation, double immunostainings were performed for CD3 (1:100, NovoCastra, UK) and Ki-67 (1:50 dilution, monoclonal, Dako, Germany) or CD20 (1:200, NovoCastra) and Ki-67, respectively. For detection of antibody binding, the REAL-HRP system (Dako REAL^TM^ Envision^TM^ Detection System, Peroxidase/DAB+, Rabbit/Mouse K5007) (for CD3 and CD20) or the REAL-AP system (Dako REAL^TM^ Detection System, Alkaline Phosphatase/RED, Rabbit/Mouse K5005) (for Ki-67) were used. Immunostained sections were then counterstained with Mayer′s Haemalaun (VWR Chemicals, Germany) and mounted.

### Cytokine Array

To measure cytokine secretion by the pituitary-infiltrating cells, single cell suspensions were prepared from pituitaries of mGH-immunized (N = 5) or CFA-immunized (N = 7) mice on day 28 post-immunization as described^19^. Cells were washed in DMEM and added to 96-well plate (1 × 10^6^/ml, 100 μl/well for three wells) in DMEM containing 10% fetal bovine serum and 100 μg/ml purified mouse growth hormone for 96 hours. Culture supernatants were 1:3 diluted and added to cytokine array membranes (RayBiotech, Norcross, GA, U. S. A.) following manufacturer’s guidelines. Fluorescent signals on the membranes were acquired by a CCD camera after addition of the detection buffers provided in the kit.

To measure cytokine secretion by splenocytes after stimulation with purified mouse growth hormone, splenocytes from mGH-immunized or CFA-immunized mice on day 28 post-immunization were isolated. Cells were stimulated by purified growth hormone as above. Cytokine secretions from the splenocytes were detected by cytokine array membranes provided by R&D Systems (Minneapolis, MN, U. S. A,).

To analyze differences in cytokine secretions in the two group of mice, signal intensities on the membranes were measured in grey scale by ImageJ software (http://imagej.nih.gov/ij/). Measured values on each spot were first subtracted from the signal in the blank spots on the membranes, then normalized to the mean measured value of positive spots on the membrane to obtain arbitrary signal units. To obtain fold change between two groups of mice, the arbitrary signal units from the mGH-immunized mice were divided by the arbitrary signal units of the CFA-immunized mice.

### Pituitary flow cytometry

To further analyze and enumerate the cell types that were proliferating in the mouse pituitary, we intraperitoneally injected 2 mg/mouse 5-bromo-2′-deoxyuridine (BrdU, eBioscience) 4 and 2 hours before sacrifice on day 28 after immunization (N = 5 per group for one experiment, total of two experiments repeated). Pooled mouse pituitaries were minced into small pieces (~1 mm^3^), and digested for 30 minutes at 37 °C in Dulbecco’s modified Eagle medium (Invitrogen) containing collagenase II (0.2% w/v, from Sigma-Aldrich). Pituitary cells from the digestion were passed through a 70-μm strainer (BD Biosciences), washed, and re-suspended in PBS, 1% BSA, 2 mM EDTA, and 0.02% sodium azide. Cells were stained first with fluorochrome-conjugated antibodies to surface markers (CD3, CD4, CD8, CD11c, CD45 and B220, all from eBioscience). Cells were then fixed, permeabilized and stained by anti-BrdU antibodies (eBioscience). Finally, cells were resuspended and analyzed by FACScalibur cytometer using CellQuest software (BD Biosciences) as described^19^.

## Additional Information

**How to cite this article**: Lin, H.-H. *et al. In Situ* Activation of Pituitary-Infiltrating T Lymphocytes in Autoimmune Hypophysitis. *Sci. Rep.* **7**, 43492; doi: 10.1038/srep43492 (2017).

**Publisher's note:** Springer Nature remains neutral with regard to jurisdictional claims in published maps and institutional affiliations.

## Supplementary Material

Supplementary Information
