# Supplementary material for: In Situ Activation of Pituitary-Infiltrating T Lymphocytes in Autoimmune Hypophysitis
Source: Sci Rep. 2017 Mar 6;7:43492. doi: 10.1038/srep43492 (PMC5337949; doi:10.1038/srep43492)

In Situ Activation of Pituitary-Infiltrating T Lymphocytes in Autoimmune Hypophysitis

Han-Huei Lin, Angelika Gutenberg, Tzu-Yu Chen, Nu-man Tsai, Chia-Jung Lee, Yu-Che Cheng, Wen-Hui Cheng, Ywh-Ming Tzou, Patrizio Caturegli and Shey-Cherng Tzou

Supplementary information

Supplementary Table 1. Diagnosis and Main Clinical Data of patients with lymphocytic hypophysitis in this study.

Patient	Age (yrs)/ gender	Presenting symptoms	Glucocorticoids before surgery	Other autoimmune diseases	Pregnancy
A[1]	33/M	9 years before operation due to sellar inflammatory granuloma, 5 years of chiasma syndrome, 6 months of visual deterioration	Yes (Prednisolone)	no	n.a.
B[1]	33/F	2 months of progredient visual deterioration, diabetes insipidus, headache and vomiting in the last months of pregnancy	no	no	3rd trimester
C[2]	32/M	4 months of headaches and visual disturbances	Yes (Prednisolone)	no	n.a.
D[2]	60/M	Over 2 yrs of bifrontal headaches	no information	no	n.a
E[2]	27/F	Secondary amenorrhea	no	no	no
F[1]	32/M	5 months of diabetes insipidus, impotence and depressions	no	no	n.a.
G[2]	56/F	12 months of headache, loss of secondary hairs, loss of libido, adynamia	no	no	no
H	62/M	7 months of headache, loss of secondary hairs	no	no	no

1 Gutenberg, A., Buslei, R., Fahlbusch, R., Buchfelder, M. & Bruck, W. Immunopathology of primary hypophysitis: implications for pathogenesis. *Am J Surg Pathol* **29**, 329-338 (2005).

2 Gutenberg, A. *et al.* Primary hypophysitis: clinical-pathological correlations. *European journal of endocrinology* **155**, 101-107, doi:10.1530/eje.1.02183 (2006).

Supplementary Table 2. Percentage of proliferating cells in the mouse pituitary gland that developed autoimmune hypophysitis.

Cell types	% of proliferating cells in the cell type
**Nonhematopoeitic cells (CD45^{-})**	0.6%
**Heamtopoeitic cells (CD45^{+})**	8.4%
T cells (CD3^{+})	11.0%
CD4^{+} T cells	5.7%
CD8^{+} T cells	10.3%
B cells (B220+)	2.9%
Dendritic cells/macrophage	3.2%

Supplementary Figure 1. Cytokine production by pituitary-infiltrating cells and splenocytes in mouse autoimmune hypophysitis. Single-cell suspensions from the pituitaries (**a**) or from the spleens (**b**) of CFA-immunized mice and mGH-immunized mice were stimulated by mGH. Cytokine production after stimulation was detected by cytokine array membranes. Signal intensity, expressed as Arbitrary Signal Units, was measured and plotted for two groups of mice. Differences in the cytokine level, expressed as Fold Change (mGH *vs.* CFA), were derived by dividing the signal intensity of mGH group by the signal intensity of the CFA group.

a

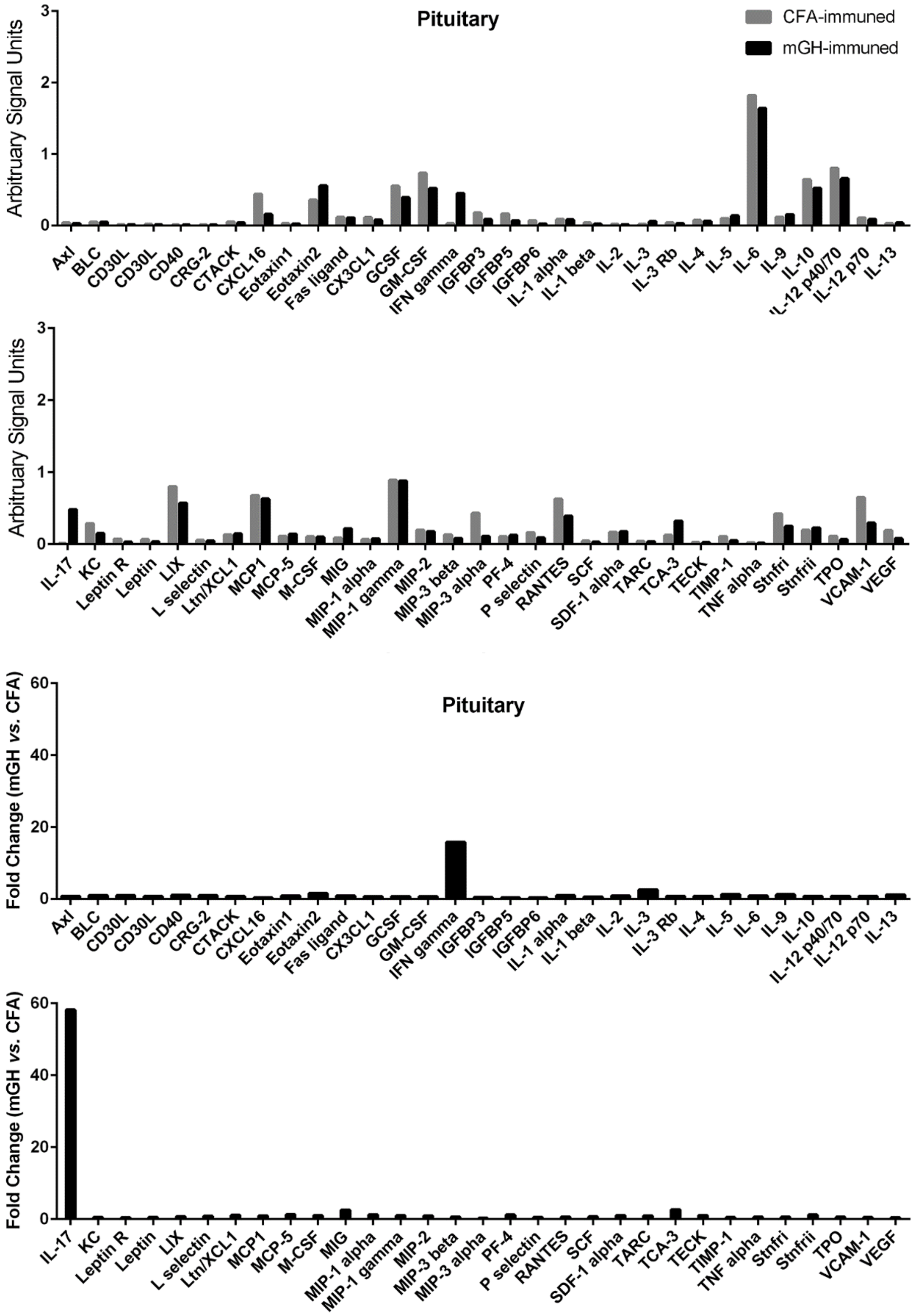
Pituitary
CFA-immuned
mGH-immuned
Arbitruary Signal Units
0
1
2
3
Axl
BLC
CD30L
CD30L
CD40
CRG-2
CTACK
CXCL16
Eotaxin1
Eotaxin2
Fas ligand
CX3CL1
GCSF
GM-CSF
IFN gamma
IGFBP3
IGFBP5
IGFBP6
IL-1 alpha
IL-1 beta
IL-2
IL-3
IL-3 Rb
IL-4
IL-5
IL-6
IL-9
IL-10
IL-12 p40/70
IL-12 p70
IL-13
Arbitruary Signal Units
0
1
2
3
IL-17
KC
Leptin R
Leptin
LIX
L selectin
Ltn/XCL1
MCP1
MCP-5
M-CSF
MIG
MIP-1 alpha
MIP-1 gamma
MIP-2
MIP-3 beta
MIP-3 alpha
PF-4
P selectin
RANTES
SCF
SDF-1 alpha
TARC
TCA-3
TECK
TIMP-1
TNF alpha
Stnfri
Stnfrii
TPO
VCAM-1
VEGF
Pituitary
Fold Change (mGH vs. CFA)
0
20
40
60
Axl
BLC
CD30L
CD30L
CD40
CRG-2
CTACK
CXCL16
Eotaxin1
Eotaxin2
Fas ligand
CX3CL1
GCSF
GM-CSF
IFN gamma
IGFBP3
IGFBP5
IGFBP6
IL-1 alpha
IL-1 beta
IL-2
IL-3
IL-3 Rb
IL-4
IL-5
IL-6
IL-9
IL-10
IL-12 p40/70
IL-12 p70
IL-13
Fold Change (mGH vs. CFA)
0
20
40
60
IL-17
KC
Leptin R
Leptin
LIX
L selectin
Ltn/XCL1
MCP1
MCP-5
M-CSF
MIG
MIP-1 alpha
MIP-1 gamma
MIP-2
MIP-3 beta
MIP-3 alpha
PF-4
P selectin
RANTES
SCF
SDF-1 alpha
TARC
TCA-3
TECK
TIMP-1
TNF alpha
Stnfri
Stnfrii
TPO
VCAM-1
VEGF

b

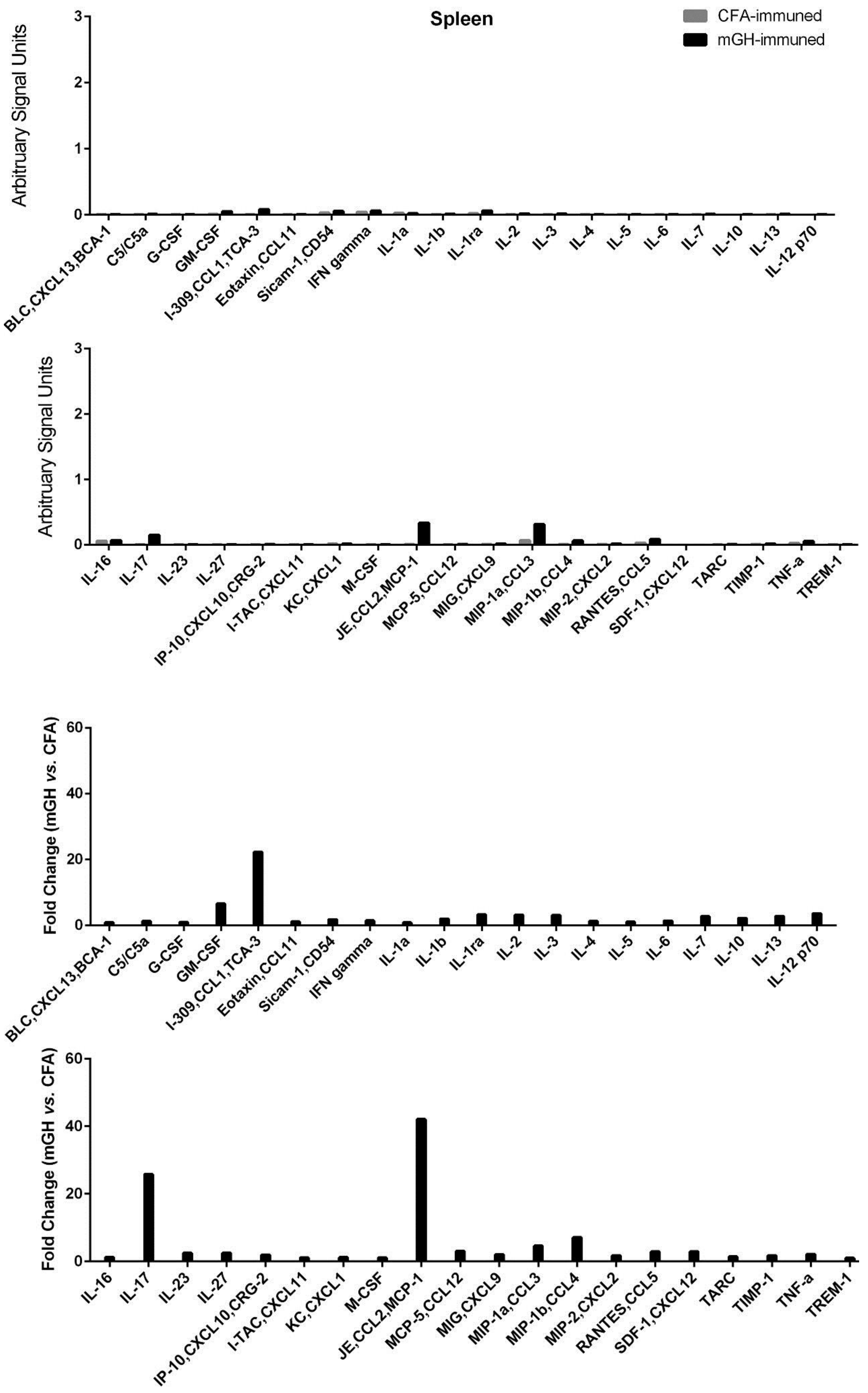
Spleen
CFA-immuned
mGH-immuned
Arbitruary Signal Units
0
1
2
3
BLC,CXCL13,BCA-1
C5/C5a
G-CSF
GM-CSF
I-309,CCL1,TCA-3
Eotaxin,CCL11
Sicam-1,CD54
IFN gamma
IL-1a
IL-1b
IL-1ra
IL-2
IL-3
IL-4
IL-5
IL-6
IL-7
IL-10
IL-13
IL-12 p70
Arbitruary Signal Units
0
1
2
3
IL-16
IL-17
IL-23
IL-27
IP-10,CXCL10,CRG-2
I-TAC,CXCL11
KC,CXCL1
M-CSF
JE,CCL2,MCP-1
MCP-5,CCL12
MIG,CXCL9
MIP-1a,CCL3
MIP-1b,CCL4
MIP-2,CXCL2
RANTES,CCL5
SDF-1,CXCL12
TARC
TIMP-1
TNF-a
TREM-1
Fold Change (mGH vs. CFA)
0
20
40
60
BLC,CXCL13,BCA-1
C5/C5a
G-CSF
GM-CSF
I-309,CCL1,TCA-3
Eotaxin,CCL11
Sicam-1,CD54
IFN gamma
IL-1a
IL-1b
IL-1ra
IL-2
IL-3
IL-4
IL-5
IL-6
IL-7
IL-10
IL-13
IL-12 p70
Fold Change (mGH vs. CFA)
0
20
40
60
IL-16
IL-17
IL-23
IL-27
IP-10,CXCL10,CRG-2
I-TAC,CXCL11
KC,CXCL1
M-CSF
JE,CCL2,MCP-1
MCP-5,CCL12
MIG,CXCL9
MIP-1a,CCL3
MIP-1b,CCL4
MIP-2,CXCL2
RANTES,CCL5
SDF-1,CXCL12
TARC
TIMP-1
TNF-a
TREM-1

Supplementary Figure 2. Lymphocytic infiltration in the pituitary gland of mouse autoimmune hypophysitis in a mid-late stage (day 56). Chronic lymphocytic infiltration developed in the pituitary after immunization with mouse growth hormone. Note the dense aggregated lymphocytic infiltration in the pituitary section.

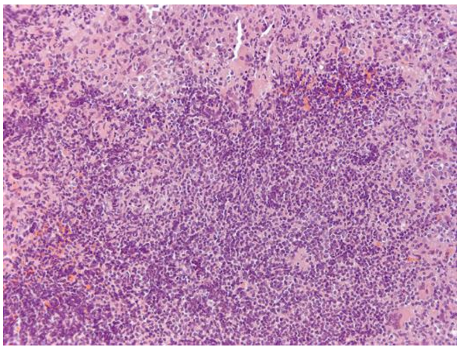

Supplementary Figure 3. Proliferation of multinucleated giant cells in the pituitary gland of mouse autoimmune hypophysitis. Multinucleated giant cells (arrow) in the pituitary sections of mice that develop autoimmune hypophysitis on day 56 were stained by PCNA (in red) but not CD3 (in blue).

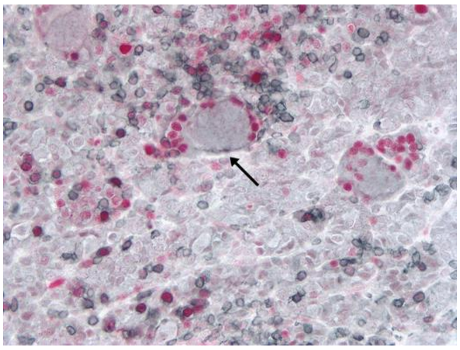

Supplement: Supplementary Information [file srep43492-s1.pdf]
